# Information Architecture for Data Disclosure

**DOI:** 10.3390/e24050670

**Published:** 2022-05-10

**Authors:** Kurt A. Pflughoeft, Ehsan S. Soofi, Refik Soyer

**Affiliations:** 1 School of Business and Economics, University of Wisconsin-Stevens Point, Stevens Point, WI 54481, USA; 2 Sheldon B. Lubar School of Business, University of Wisconsin-Milwaukee, Milwaukee, WI 53211, USA; esoofi@uwm.edu; 3 Department of Decision Sciences, The George Washington University, Washington, DC 20052, USA; rsoyer@gwu.edu

**Keywords:** data confidentiality, data utility, differential privacy, disclosure risk, Kullback–Leibler information, maximum entropy

## Abstract

Preserving confidentiality of individuals in data disclosure is a prime concern for public and private organizations. The main challenge in the data disclosure problem is to release data such that misuse by intruders is avoided while providing useful information to legitimate users for analysis. We propose an information theoretic architecture for the data disclosure problem. The proposed framework consists of developing a maximum entropy (ME) model based on statistical information of the actual data, testing the adequacy of the ME model, producing disclosure data from the ME model and quantifying the discrepancy between the actual and the disclosure data. The architecture can be used both for univariate and multivariate data disclosure. We illustrate the implementation of our approach using financial data.

## 1. Introduction

Preserving confidentiality of individuals is an important issue for both public and private agencies that have the responsibility to share data with the public. On one hand, public agencies such as the Federal Housing Association (FHA) or private institutions such as banks, hospitals, and insurance companies are expected to share the data they have for the purposes of data analysis and research, but at the same time they are required to protect the privacy of the individuals. Both the public and private agencies owning such data are faced with the challenge of determining how to release them for public use. This problem is referred to as the data disclosure problem.

The data disclosure problem is one aspect of the several issues of the general problem of preserving confidentiality in data analysis. It comes about because in certain societies, notably the U.S., data that is gathered using taxpayer resources has to be made available to the public, but under the caveat that the released data does not betray public trust vis a vis a compromise of confidentiality. As a consequence, government agencies strike a balance by “masking” the data prior to its release, but in a manner that endeavors to preserve the essential information that the data contains. This is because the released data is often used to make public policy decisions in areas such as economics, finance, health, housing and trade, to name a few. The driving motto here is that the released data should contain a certain amount of the truth, but not the whole truth. The essence of the problem therefore boils down to determining how much of the truth should be revealed and how the rest should be concealed so that there is a fair balance between the data’s value (or utility) to a legitimate user and the data’s confidentiality. These issues are not limited only to public institutions but are also relevant for private organizations in financial, health and insurance industries that deal with confidential data on a day-to-day basis. For example, Franconi and Stander [1] and Ichim [2] discuss data disclosure issues for business microdata.

To implement the paradigm of calculated disclosure, a plethora of approaches have been proposed in literatures of various disciplines including computer science, statistical science, management science and decision science. An overview of some of these literatures is given in the sequel. By and large, many of the proposed approaches are, de facto, purely statistical in nature, whereas a handful have a decision-theoretic character. Of the latter, some have an information-theoretic basis and the focus of this paper falls in this general category. A complete treatment of statistical confidentiality and related topics can be found in the book by Duncan et al. [3]. Recent advances on data privacy and confidentiality are discussed in the special issue edited by Liu et al. [4].

The main challenge in the data disclosure problem is to release data such as to avoid misuse by intruders while providing useful information to legitimate users for analysis. Duncan and Lambert [5] point out that disclosure can be of three types: Identity disclosure (i.e., identifying a respondent from the released data), attribute disclosure (i.e., obtaining information about a respondent from the released data), and inferential disclosure (i.e., deducing new information about a respondent from the released data). Thus, as noted by Kadane et al. [6], an individual’s identity can be revealed by linking, matching or looking for unique characteristics in the released data.

An approach to warding off the above obstacles is to mask the data before release. Fienberg [7] characterizes data masking as “The disclosure limitation process of transforming a data set when there is a functional relationship (possibly stochastic) between the masked values and the original data”. Data masking strategies tend to be statistical, such as releasing a sample of the data, including simulated data in the original data, excluding certain attributes, perturbing the data with noise, swapping the data, releasing only simulated data, etc.; see for example, [8,9,10,11,12,13,14,15]. Fienberg [7] classifies the above methods as suppression, recoding, sampling and simulation. A detailed review of these methods can be found in [16] and a more recent discussion is given in [17].

Data masking cannot ensure confidentiality with certainty. As a consequence, “agencies that release masked data try to maintain an acceptable disclosure risk level rather than a zero risk” [5]. Thus, the focus of recent work is on developing methods for limiting disclosure risk and balancing the trade-off between confidentiality and data utility. For example, Hu et al. [18] considered utility-risk trade-off in the release of microdata. As a result, a consideration of both the decision theoretic as well as the statistical approaches for addressing the data disclosure problem have become germane. Since decision theoretic procedures entail the use of utility functions, some of the literature in this area pertains to a discussion of meaning of utility functions. For example, Karr et al. [19] define and compare data utility in terms of the Kullback–Leibler (KL) divergence between the parametric empirical distributions of the original and the released data. In others, such as the paper by Keller-McNulty et al. [20], data utility is specified in terms of entropy. Sankar et al. [21] also considered information theoretic measures to describe utility-risk trade-off.

Trottini [22,23] provided a comprehensive decision-theoretic approach for balancing the trade-off between preserving confidentiality and data utility. In recognition of the fact that maximizing confidentiality and maximizing data utility are conflicting objectives, the author formalizes the problem using a multiattribute utility theory framework in the sense of Keeney and Raiffa [24]. In so doing, he focuses on discrete tabular data and proposes a decision-theoretic framework for developing data releasing strategies for different levels of confidentiality and data utility. Trottini’s measure of data utility is the closeness of the masked data to the original data and his approach for balancing the trade-off between data utility and confidentiality entails a use of multiattribute utility theory. Trottini’s work is conceptual; for example, there is no specification of the utility of confidentiality as considered by Keller-McNulty et al. [20]. A detailed review on data utility and disclosure risk is given by Cox et al. [25] where the authors point out the ambiguity involved in definition of these two concepts as well as their measurement.

A concept related to disclosure risk is the differential privacy (DP) standard proposed originally by Dwork [26] which has gained recent attention in the literature; see [27]. Dwork [26] defines DP as “… differential privacy ensures that the removal or addition of a single database item does not (substantially) affect the outcome of any analysis”. As noted by Snoke and McKay-Bowen [27], DP offers only a partial solution to the data disclosure problem; that is, it provides a “rigorous definition of privacy loss”. As a result, the DP “methodology has drawbacks when it comes to preserving the utility of the data or carrying out valid statistical inference”.

Polettini [28] describes a maximum entropy (ME) approach for arriving upon a distribution from which the data to be released can be simulated. Polettini’s approach is geared towards determining the ME distribution which retains the key information moment features of the data. Polettini [28] does not include data on continuous variables and does not address the issue of compatibility of the ME distribution with the actual data.

The present paper proposes an information architecture that provides a comprehensive framework for the data disclosure problem. The main contribution of the paper is a novel and systematic integration of some information-theoretic ideas that have appeared in the data disclosure literature along with utilizing an Euclidean statistical measure which thus far has not been used for the disclosure problem. The architecture extends: (a) Polettini’s [28] work to the full force of ME modeling as is articulated in [29], (b) Karr et al.’s [19] use of the Kullback–Leibler divergence between the multivariate normal models for the actual and disclosure data to the full force of the information divergence between any pair of probability distributions, and (c) Keller-McNulty et al.’s [20] idea of using an entropy-based measures to describe data utility to the use of information divergence and Euclidean measures for assessing the utility/risk of the data disclosure. The proposed approach addresses the utility-risk trade-off inherent in the data disclosure without suffering from the drawbacks of the DP based methods. Furthermore, the use of ME models, which are parametric, for generating release data, enables us to avoid increased privacy risks associated with nonparametric approaches; see Awan et al. [30]. However, we utilize parametric, semi-parametric and nonparametric Euclidean measures for evaluating the chosen ME model and the generated disclosure data. Step-by-step implementation of the proposed architecture is described and illustrated by application to two financial data sets.

The paper is organized as follows. Section 2 describes the information architecture for the data disclosure problem. Section 3 presents the essentials of ME modeling and measures used for implementing the architecture. Section 4 illustrates the architecture using two financial data sets, mortgage default data and bank accounts data. Concluding remarks are given in Section 5. An Appendix A tabulates examples of univariate and bivariate ME models and their information moments.

## 2. Information Architecture

The information architecture for data disclosure is based on the view that the data is generated according to an unknown distribution. We denote the cumulative distribution function (CDF) of the data-generating distribution by *F*, its probability density function (PDF) relative to a measure ν by *f* and the associated random vector by $X=(X_{1},\ldots ,X_{p})$. We assume minimal information about *f* in terms of the following class of distributions:

(1) $$\Omega =\{f:E_{f}\left[T_{j}\left(X\right)\right]=\theta_{j},\;j=1,\cdots ,J\}.$$

The set of information moments, $T=\{T_{1},\ldots ,T_{J}\}$, are also unknown and has to be explored from the data.

The architecture combines two basic elements of statistics with two basic elements of information theory. The exploratory data analysis promoted by Tukey in 1970s uses statistical graphics to summarize main characteristics of the data distribution. Probability models are produced for the unknown data-generating distribution to infer about the reproducibility of essentially similar data. The entropy of a probability distribution, information divergence between probability distributions and distance between two data sets are three basic elements of the proposed architecture. Combining the statistical and information-theoretic elements serves the purpose of learning from data $x_{i}=\left(x_{1i},\ldots ,x_{pi}\right),i=1,\ldots ,n$ to produce a sample of *n* secure data points for disclosure, $x^{*}=\left(x_{1i}^{*},\ldots ,x_{pi}^{*}\right)$. For example, in our first application $x_{i}=\left(x_{1i},x_{2i}\right)$ are data on two sensitive items, the amount of the mortgage loan and the income of the borrower, which must be protected from intruders who want to identify individuals.

The architecture produces a statistical replica $x^{*}$ from a probability model $F^{*}$ for *F* with PDF $f^{*}$ and includes multiple inspections for checking accuracy of the model and replica. The architecture aims to produce disclosure data with the following properties:

(a) The essential statistical aspects, such as underlying distribution and information moments of the actual and disclosure data sets are about the same.
(b) The individual points in the actual and disclosure data sets are not similar.

**Figure 1:**
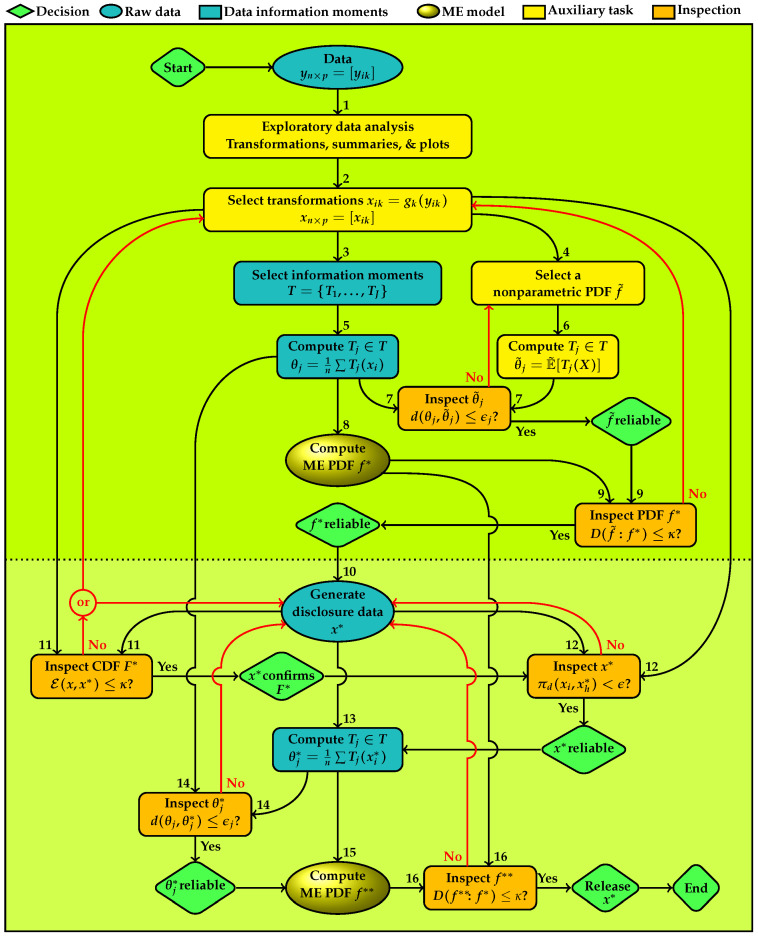
Plan of the data disclosure; numbers indicate sequence of tasks; *d* is Euclidean distance; *D* is information divergence; E is energy statistic; π is proportion of distances between all possible pairs of points in the actual and disclosure data.

Figure 1 depicts the plan of the information architecture with sixteen tasks which are enumerated to be completed sequentially, and six decision points with “Yes” and “No” outcomes are shown. Tasks in the upper panel are for developing a reliable ME model for the data and those in the lower panel are for developing reliable disclosure data. Tasks shown in the column under the node for data are for the ingredients of ME models and producing disclosure data. All other tasks are for checking accuracy. Colors and shapes of nodes group types of the tasks. Green rhombuses are decision nodes. Blue ellipses show the actual and disclosure data. Blue rectangles are for information moments and data summaries that provide ME models. The ME PDFs are highlighted in golden ellipses. Yellow rectangles are tasks that provide materials for quality control which are shown in orange rectangles.

**Task 1** 
starts the process with an exploratory data analysis of the raw data. Various distribution plots and scatter plots are produced for the following purposes.
  (a) To explore the distributional features of the data that provide clues for selecting a transformation for specifying the set of information moments *T* for an ME model $f^{*}$, as a parametric representation of *f*, which is used for generating a replica for disclosure.
  (b) To explore a suitable nonparametric PDF $\tilde{f}$ that represents *f* for checking the adequacy of $f^{*}$.
**Task 2** 
specifies transformations of the original data y to x, where $x=g_{k}\left(y\right)$, hereafter is called the actual data, k=1,…,p are one-to-one functions on *ℜ* which transforms the coordinates of y. The identity function $g_{k}\left(y_{k}\right)=y_{k}$ is included when a transformation is not needed.
**Task 3** 
specifies the set of information moments for deriving the parametric ME model $f^{*}$ to represent *f*.
**Task 4** 
provides a nonparametric PDF $\tilde{f}$, for representing *f*. For continuous variables, $\tilde{f}$ is a multivariate kernel density estimate or histogram. For the discrete and categorical variables, $\tilde{f}$ is the distribution of relative frequencies. This distribution serves as an intermediary for examining suitability of the information moments for developing an ME model for the data. This mediation is necessary for the continuous variables because the information moments of the raw data given in (2) are based on the usual empirical distribution, which does not possess a continuous PDF for confirming a continuous ME model.
**Task 5** 
computes the specified moment information. For example, equal weights of data points give
  (2) $$\theta_{j}=\frac{1}{n}\sum_{i=1}^{n}T_{j}\left(x_{i}\right),\;j=1,\ldots ,J.$$
  These information moments can include usual moments such as various power and cross-product moments, quantiles such as median where $T_{j}\left(x\right)$ is an indicator function, and/or more complex type such as those shown in Table A1, Table A2 and Table A3 in the Appendix A [29,31]. In the case of frequency tables, $T_{j}\left(x\right),j=1,\ldots ,J$ represent univariate and multivariate marginal frequencies of contingency tables. The information architecture for disclosure accomplishes data protection via creating a statistical copy $x^{*}$ of x for disclosure, both of which possess approximately the same information moments as the actual data.
**Task 6** 
computes the information moments of $\tilde{f}$ given by
  (3) $${\tilde{\theta }}_{j}=\int T_{j}\left(x\right)\tilde{f}\left(x\right)d\nu \left(x\right),\;j=1,\ldots ,J;$$
  for continuous variables dν(x)=dx and for discrete variables dν(x)=1 and the integral changes to summation. The idea is that if $\tilde{f}$ is a good representation of the information characteristics of the data then its information moments should be approximately the same as those given in (2).
**Task 7** 
has two input links to inspect the Euclidean distance $|\theta_{j}-{\tilde{\theta }}_{j}|$ between each information moment of the nonparametric PDF and the corresponding data information moment. If any ${\tilde{\theta }}_{j}$ is not confirmed, $\tilde{f}$ has to be revised and the information moments of the revised $\tilde{f}$ should be examined. The revision can include, for example, changing the grid used for computing the information moments and the bandwidth of the kernel density, type of the kernel function, the bins of histogram or the type of empirical PDF. If all individual ${\tilde{\theta }}_{j}$’s are confirmed, then the empirical PDF is reliable for using to inspect the adequacy of the ME model for the data. The first decision node shown at the right side of this node in Figure 1) displays this conclusion.
**Task 8** 
computes the ME model for x, shown as $f^{*}$, implied by the set of data information moments $\left\{\theta_{j},j=1,\ldots ,J\right\}$.
**Task 9** 
has two input links to inspect the information divergence between the multivariate PDF of the ME model, $f^{*}$ and the nonparametric PDF that represents the data. The multivariate divergence examines entire set of moments *T* and lower dimensional divergence measures examine respective subsets of marginal information moments. This task serves two purposes.
  (a) The information divergence measure between two distributions is inclusive of all information moments of reflected of $\tilde{f}$ and $f^{*}$, hence provides an aggregate measure of discrepancy between their sets of moments.
  (b) The information divergence examines the adequacy of the ME PDF for representing the nonparametric PDF of the data.
  If $f^{*}$ is not confirmed, then selection of information moments has to be revised for which revisiting data exploratory analysis becomes necessary. The revision can include reexamining transformations, selection of the information moments and the nonparametric PDF. Upon the revision, all preceding nodes have to be revisited. If $f^{*}$ is confirmed, the role of $\tilde{f}$ ends. We conclude that the information moments $\left\{T_{j},j=1,\ldots ,J\right\}$ represent the statistical characteristics of the data. By the Entropy Concentration Theorem (Jaynes [32]), if the data generating distribution is governed by the selected information moments, then the ME distribution closely approximates types of distributions that satisfy the moments. This property makes $f^{*}$ reliable for inferential purposes. The second decision node shown below ME model in Figure 1 displays this conclusion.
  Then the process proceeds with using the ME model for generating disclosure data.
**Task 10** 
uses $f^{*}$ to generate the statistical copy $x^{*}$ for disclosure, which will be subject to four inspections for approval to release.
**Task 11** 
uses $x^{*}$ to reaffirm the ME model via the energy statistic $E(x,x^{*})$ which measures the difference between two distributions based on the pairwise Euclidean distance on ${ℜ}^{p}$, defined by
  (4) $$d\left(x_{i},z_{h}\right)=\left|x_{i}-z_{h}\right|,\;\mathrm{for}\;\mathrm{all},\;i,h=1,\ldots ,n.$$
  Distances between data points in the actual and disclosure data sets, $d(x_{i},x_{h}^{*})$, are assessed in terms of the difference between their average and the average of distances within each data set $d(x_{i},x_{h})$ and $d(x_{i}^{*},x_{h}^{*})$. For measuring the model fit $E(x,x^{*})$ should be low. If the value of $E(x,x^{*})$ is not negligible, a new set of data has to be generated and reexamined. If regeneration does not produce a satisfactory result, selection of information moments has to be revised for which revisiting data exploratory analysis becomes necessary. Upon the revision, all preceding nodes must be revisited. If the ME model is confirmed, the process continues with further inspections of the disclosure data. The third decision node shown below the disclosure data in Figure 1 displays this conclusion.
**Task 12** 
inspects the proportion of distances between the points in the actual and disclosure data,
  (5) $$\pi_{d}\left(x_{i},x_{h}^{*}\right)=\frac{1}{n^{2}}\sum_{i=1}^{n}\sum_{h=1}^{n}I\left(d\left(x_{i},x_{h}^{*}\right)\leq d_{0}\right)<\epsilon ,$$
  where I(A) is the indicator function of condition *A*. This measure is used for controlling the disclosure risk of $x^{*}$. The disclosure data is synthetic, generated from the ME model for the actual data. There is not a one-to-one correspondence between the points in the two data sets. However, there still can be a disclosure risk, for example, when each disclosure data point is very close to an actual data point. If $\pi_{d}\left(x_{i},x_{h}^{*}\right)$ does not produce a satisfactory result, a new set of disclosure data ought to be generated and reexamined. If $x^{*}$ is confirmed, the fourth decision node reflects this conclusion and the process continues with the information moments.
**Task 13** 
computes the information moments $T_{j}\in T$ of the disclosure data, denoted as $\theta_{j}^{*}$. As noted before, *T* includes marginal and joint moments of various types.
**Task 14** 
uses two input links for $|\theta_{j}-\theta_{j}^{*}|$ to inspect each information moment of the release data with the corresponding information moment of the actual data. If the closeness of the pairs of all information moments is not confirmed, a new version of disclosure data has to be generated and reexamined through Tasks 11–14. If $\theta_{j}^{*}$ are confirmed individually, then the set of disclosure data moments $\left\{\theta_{j}^{*},j=1,\ldots ,J\right\}$ is reliable. The fifth decision node at the east of this node in Figure 1 displays this conclusion and the process proceeds with computation of the ME model for the disclosure data for further inspections.
**Task 15** 
computes the ME model $f^{**}$ implied by the set of data information moments $\left\{\theta_{j}^{*},j=1,\ldots ,J\right\}$ for the inspection the entire set as a whole.
**Task 16** 
serves the purpose of examining the information discrepancy between $f^{**}$ and $f^{*}$. The multivariate divergence examines entire *T* and the marginal divergence measures examines subsets of marginal information moments. If the closeness of the two ME models is not confirmed, a new set of data has to be generated and reexamined through Tasks 11–16. With approval of $f^{**}$ the sixth decision node in southeast corner of Figure 1 displays the following conclusion: $x^{*}$ is a statistical replica of x and ready for disclosure. Then the process ends.

## 3. Implementation of the Information Architecture

### 3.1. ME Information Moments

The exploratory data analysis task of the information architecture provides a variety of plots and summary measures that reveal various distributional aspects of the data. These tools provide evidence about specifying a set of potential information moments that lead to a model for the data distribution.

The ME model in (1) is defined by $f^{*}$ that maximizes Shannon entropy,

(6) H(X)=H(f)=−∫f(x)logf(x)dν(x),

provided that the integral is finite. If $f^{*}\in \Omega$ exists, it is unique and is in the following form

(7) $$f^{*}\left(x\right)=C\left(\lambda \right)e^{-\lambda_{1}T_{1}\left(x\right)-\cdots -\lambda_{J}T_{J}\left(x\right)},$$

where the vector of natural parameters $\lambda =(\lambda_{1},\cdots ,\lambda_{J})$ consists of the Lagrange multipliers determined by the moment information constraints in (1) and

(8) $$C\left(\lambda \right)={\left[\int e^{-\lambda_{1}T_{1}\left(x\right)-\cdots -\lambda_{J}T_{J}\left(x\right)}d\nu \left(x\right)\right]}^{-1}$$

is the normalizing factor of the PDF. The ME model exists if the integral in (8) is finite. The entropy of the ME model (7) is

(9) $$H\left(f^{*}\right)=-\log C(\lambda )+\sum_{j=1}^{J}\lambda_{j}\theta_{j}.$$

Any distribution with finite entropy can be characterized as the ME model in a class of distributions which can be identified by representing its density in the form of (7) [29]. These authors give an example which underscores the importance checking the finiteness of H(f). Several examples of ME models and information moments are listed in Table A1 of Appendix A.

Transformation of data facilitate the search for information moments. In general, a transformation decreases entropy [33]. In the discrete case, entropy is invariant under one-to-one transformations, but the continuous entropy is not invariant under all one-to-one transformations. Entropy transformation formula is available, see [29,34]. Aulogiaris and Zografos [35] and Zografos [36] have used the relationship $H\left(F_{Y}\right)=H\left(F_{X}\right)+\log\left|A\right|$ for the affine transformation Y=AX+b, |A|≠0 to deduce the relationship between characterizations of $F_{Y}^{*}$ and $F_{X}^{*}$ for some particular distributions. H(X) is invariant under translation and under orthonormal transformation, H(AX)=H(X), where *A* is d×d matrix with determinant |A|=1. Ebrahimi et al. [29] provided a result for identifying the class of distributions where the distribution of an arbitrary one-to-one transformation of X is the ME model. Examples of transformations of several ME models included in Table A1 are shown in Table A2 of Appendix A. A formula for computing entropy of transformed variable is available, see for example, [29].

Information measures are functional of PDFs which apply to the multivariate case as well. Marginal distributions of a multivariate distribution can be in the same family or in different families. Table A3 gives examples of varieties of bivariate ME models. The bivariate normal, Pareto, Farlie-Gumbel-Morgenstern (F-G-M), Dirichlet distributions extends to multivariate case. McKay’s bivariate gamma is an example where both marginal distributions are gamma while the support of one is bounded below by the other. The gamma–gamma distribution is an example where the marginal distributions are in a different family.

More generally, univariate distributions in the same and different families can be joined through various link functions to form multivariate distributions (see, for example, [37]). A widely used such method is through a copula. The use of copula in data disclosure problem is discussed in [38]. The copula of a bivariate distribution *F* is defined by

(10) $$C\left(u_{1},u_{2}\right)=F\left(F_{1}^{-1}\left(u_{1}\right),F_{2}^{-1}\left(u_{2}\right)\right),\;u_{k}\in \left[0,1\right],$$

where $f_{k}\left(u\right)=1$. The CDFs of normal and F-G-M PDFs shown in Table A3 are well-known as Gaussian and F-G-M copulas. The survival copula is defined similarly in terms of a bivariate survival function. The survival function of Pareto PDF shown in Table A3 is well-known as Clayton copula.

### 3.2. Discrepancy Measures

The information architecture depicted in Figure 1 uses the information divergence for assessing model adequacy and Euclidean distance for the discrepancy between the actual and disclosure data sets. Various discrepancy measures can be used for these purposes. We describe our preferred measures.

#### 3.2.1. Energy Statistic

The squared energy distance between the CDFs of the distribution that generated the actual data x and the CDF of the ME distribution that we used to generate the disclosure data $x^{*}$ is defined by

(11) $$D^{2}\left(X,X^{*}\right)=2E|X\;-\;X^{*}|\;-\;E|X\;-\;X_{c}\left|\;-\;E\right|X^{*}\;-\;X_{c}^{*}|\;\geq \;0,$$

where X and $X_{c}$ are identically distributed as *F*; the inequality becomes an equality if and only if $F=F^{*}$ The energy statistic is defined by the empirical version of (11); see Rizzo and Székely [39] for a review of energy distance/statistic.

Baringhaus and Franz [40] proposed a nonparametric statistic in terms of the empirical version of (11) for testing the equality of two CDFs. Their test for two sets of equal size *n* is given by

(12) $$E\left(x,x^{*}\right)=\frac{1}{n}\left(\sum_{i=1}^{n}\sum_{h=1}^{n}\left|x_{i}-x_{h}^{*}\right|-\frac{1}{2}\left[\sum_{i=1}^{n}\sum_{h=1}^{n}|x_{i}-x_{h}|+\sum_{i=1}^{n}\sum_{h=1}^{n}\left|x_{i}^{*}-x_{h}^{*}\right|\right]\right)\geq 0,$$

where the inequality becomes an equality if and only if $F=F^{*}$. This statistic measures the discrepancy between two distributions by the average Euclidean distance between points in the two samples as compared with the averages of distances between points within each data set. As such, this test is analogous to the analysis of variance in statistics.

Baringhaus and Franz [40] called (12) Cramér statistic, apparently as a multivariate extension of the Cramér-von Mises statistic. We call (12) as energy statistic due to the fact that it is n/2 times the energy statistic between two equal size samples.

#### 3.2.2. Kullback–Leibler Information Divergence

The basic information divergence between two distributions is the Kullback–Leibler (KL) divergence (relative entropy) defined by

(13) $$K\left(f_{1}:f_{2}\right)=\int f_{1}\left(x\right)\log\frac{f_{1}\left(x\right)}{f_{2}\left(x\right)}d\nu \left(x\right)\geq 0,$$

provided that $f_{2}\left(x\right)=0$ only if $f_{1}\left(x\right)=0$ (absolute continuity condition). The inequality becomes an equality if and only if $f_{1}\left(x\right)=f_{2}\left(x\right)$, almost everywhere.

The statistical information aspect of $K(f_{1}:f_{2})$ is rooted in Bayes theorem. Let $M_{1}$ and $M_{2}$ be two models that specify $f_{1}\left(x\right)=f\left(x|M_{1}\right)$ and $f_{2}\left(x\right)=f\left(x|M_{2}\right)$ for the distribution of *X* with prior probabilities $P(M_{1})$ and $P(M_{2})$. Then, by Bayes theorem, $K(f_{1}:f_{2})$ is the expected difference between the posterior and prior log-odds in favor of $M_{1}$ against $M_{2}$ (Kullback and Leibler [41]).

In the information architecture this measure serves for examining the discrepancy between the nonparametric PDF $\tilde{f}$ and the ME PDF $f^{*}$ (Task 9) and the discrepancy between the PDF of the ME model for the data $f^{*}$ and the PDF of the ME model for the disclosure data $f^{**}$ (Task 16). The KL divergence can be used for two multivariate PDFs, as well as for the univariate case. $K(f_{1}:f_{2})$ is not symmetric. However, symmetrized versions of it, such as Jeffreys divergence, are available in the literature. An important property of the KL divergence is invariance under the one-to-one transformations, allowing us to implement inspections of the ME models in the information architecture in terms of $x_{i}=g_{k}\left(y_{i}\right)$.

The information index of two continuous PDFs is defined by the normalized information measure,

(14) $$\delta \left(K\right)=1-e^{-2K(f:f^{*})},\;0\leq \delta^{2}\left(K\right)\leq 1,$$

where values close to zero imply that the ME model provides a good fit *f*. McCulloch [42] defined a calibration in terms of the difficulty of discrimination between a fair and a biased coin with probability q≥0.5, where

(15) $$q\left(K\right)=0.5[1+\delta^{2}{\left(K\right)}^{1/2}].$$

The independence between random variables is defined by the condition where their joint distribution factors into the product of their marginal distributions. It is well known that the association indices such as the product moment correlation, Spearman’s rank correlation and Kendall’s tau fail to reveal various forms of dependence [43]. Dependence is measured by the divergence between the joint distribution and the independent model. In the information architecture dependence between variables is measured by the mutual information defined by the KL divergence between the joint PDF and the product of marginal PDFs. For a bivariate distribution *f* with marginals $f_{k},k=1,2$ the mutual information is defined by

(16) $$\begin{array}{ccc} M\left(f\right)=M\left(X_{1},X_{2}\right) & = & K(f:f_{1}f_{2}) \end{array}$$

(17) $$\begin{array}{ccc} & = & H\left(f_{1}\right)+H\left(f_{2}\right)-H\left(f\right). \end{array}$$

$M(X_{1},X_{2})\geq 0$, where the equality holds if and only if the variables are independent. The information index of dependence is

(18) $$\delta^{2}\left(M\right)=1-\exp\left\{-2M\right\}.$$

For a multivariate distribution, various mutual information measures of dependence are available. For example,

(19) $$\begin{array}{c} M\left(X_{1},X_{2},X_{3}\right)=H\left(f_{1}\right)+H\left(f_{2}\right)+H\left(f_{3}\right)-H\left(f\right) \end{array}$$

(20) $$\begin{array}{c} M\left[X_{1},\left(X_{2},X_{3}\right)\right]=H\left(f_{1}\right)+H\left(f_{23}\right)-H\left(f\right). \end{array}$$

### 3.3. Semi-Parametric Measures

Computations of the information moments of the nonparametric PDF $\tilde{f}$ and the information divergence $K(\tilde{f}:f^{*})$ are based on numerical integration which is implemented on a grid. Consider a bivariate application problem. Partition the support by a set of grid points:

(21) $$\{\left(\xi_{1a},\xi_{2b}\right)\},\;a=0,\ldots ,A,\;b=0,\ldots ,B,$$

where $\left(\xi_{10},\xi_{20}\right)=\inf\left\{\left(x_{1},x_{2}\right):F\left(x_{1},x_{2}\right)\approx 0\right\}$, $\left(\xi_{1A},\xi_{2B}\right)=\inf\left\{\left(x_{1},x_{2}\right):F\left(x_{1},x_{2}\right)\approx 1\right\}$. Let the increments for each variable remain constant denoted by $w_{1}=\xi_{1(a+1)}-\xi_{1a},$ $w_{2}=\xi_{2(b+1)}-\xi_{2b},a=0,\ldots ,A,b=0,\ldots ,B$. Then the trapezoid numerical integration of the bivariate nonparametric density $\tilde{f}$ gives

(22) $$\tilde{P}\left(X_{1}\in w_{1},X_{2}\in w_{2}\right)\approx \tilde{f}\left(x_{1},x_{2}\right)w_{1}w_{2},$$

which on a countable partition may not sum to one, exactly. The normalized probabilities are obtained by

(23) $${\tilde{P}}_{ab}\approx \frac{\tilde{f}\left(\xi_{1a},\xi_{2b}\right)}{\sum_{a=1}^{A}\sum_{b=1}^{B}\tilde{f}\left(\xi_{1a},\xi_{2b}\right)}.$$

The information moments of $\tilde{f}$ are approximated by

(24) $${\tilde{\theta }}_{j}\approx \sum_{a=1}^{A}\sum_{b=1}^{B}T\left(\xi_{1a},\xi_{2b}\right){\tilde{P}}_{ab}.$$

The approximate probability under $f^{*}$, denoted by $P_{ab}^{*}$, are computed similarly as in (23) and the KL divergences between $\tilde{f}$ and $f^{*}$ is computed on the grid as follows:

(25) $$K\left(\tilde{f}:f^{*}\right)\approx \sum_{a=1}^{A}\sum_{b=1}^{B}{\tilde{P}}_{ab}\log\frac{{\tilde{P}}_{ab}}{{\tilde{P}}_{ab}^{*}}$$

The mutual information of a nonparametric PDF can be approximated either by (16) or by approximating entropies in (17). The entropy of $\tilde{f}$ can be approximated by the histogram entropy estimate of [44] given by

(26) $$H\left(\tilde{f}\right)\approx -\sum_{a=1}^{A}\sum_{b=1}^{B}{\tilde{P}}_{ab}\log{\tilde{P}}_{ab}+\log\left(w_{1}w_{2}\right)$$

The marginal entropies in (17) can be approximated using the marginal distributions of the bivariate distribution $\tilde{P}=\left[{\tilde{P}}_{ab}\right]$.

## 4. Disclosure of Financial Data

Financial data such as income, loan amount, bank account balance and deposits provide highly sensitive information. The distributions of these variables are generally skewed and in some cases are heavy tailed. The large values of these variables are easily identifiable. Addition of noise to the data retains these characteristics. In what follows, we use two examples to illustrate applications of the proposed information architecture.

### 4.1. Mortgage Data

Soyer and Xu [45] considered mortgage default data provided by FHA. The data consists of 400 observations on sensitive variables such as the income of the individual ($Y_{1}$) and the loan amount ($Y_{2}$). These variables are important in modeling the time to default of mortgages, but they also carry a risk of disclosure. Implementation of the information architecture is as follows.

#### 4.1.1. Exploratory Analysis

**Figure 2:**
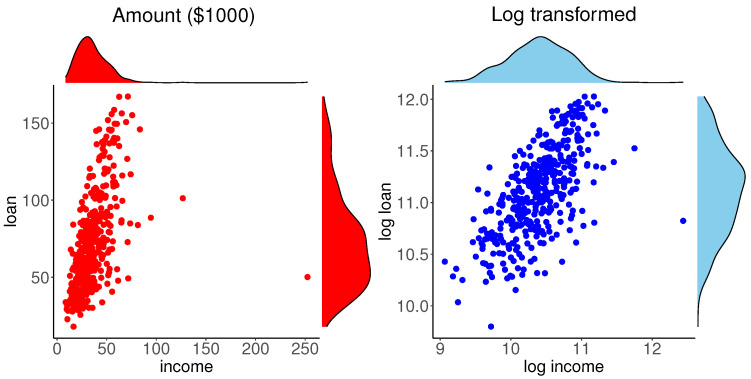
Plots of original and log transformed mortgage data.

Our exploratory analysis of this data is depicted in Figure 2. The left panel shows the scatter plot of the data with the marginal PDFs obtained by the kernel density estimate. A high-income data point is clearly identifiable and the marginal distributions are highly skewed. The marginal and bivariate plots data suggest considering logarithm transformation of the variables, which is common in analysis of financial data. The scatter plot with the marginal PDFs for log transformed data are shown in the right panel of Figure 2. This scatter plot is nearly elliptical, except for an outlying point and the PDFs are nearly symmetric. These plots suggest considering the bivariate normal distribution for $\left(x_{1},x_{2}\right)=\left(\log y_{1},\log y_{2}\right)$.

#### 4.1.2. Information Moments and ME Model

Table A3 gives the information moments as the first two marginal means and the cross-product moment of the logarithm of the two variables. The selected information moments are

(27) $$\begin{array}{c} \mu_{k}=\int x_{k}f\left(x\right)dx,\;k=1,2, \end{array}$$

(28) $$\begin{array}{c} \sigma_{kh}=\int \left(x_{k}-\mu_{k}\right)\left(x_{h}-\mu_{h}\right)f\left(x\right)dx,\;k,h=1,2. \end{array}$$

The ME model for the transformed variables is the bivariate normal with PDF $f^{*}\left(x\right)=N\left(\mu ,\Sigma \right)$, where

(29) $$\mu =\left[\begin{array}{c} \mu_{1}\\ \mu_{2} \end{array}\right],\;\Sigma =\left[\begin{array}{cc} \sigma_{11} & \rho \sigma_{1}\sigma_{2}\\ \rho \sigma_{1}\sigma_{2} & \sigma_{22} \end{array}\right],\;\rho =\frac{\sigma_{12}}{\sigma_{1}\sigma_{2}},\;\sigma_{k}=\sqrt{\sigma_{kk}}.$$

Table 1 reports univariate and bivariate statistics for implementing the tasks shown in the upper panel of Figure 1. The first column gives the information moments of the log-transformed data and the second column gives the corresponding parameters of the kernel estimate. The corresponding measures are close. Theoretically, the mean of a kernel PDF is the same as the sample mean, however the variance and covariance include correction terms as follows:

(30) $$\sigma_{kj}=v_{kj}+h_{k}h_{j}\kappa_{2},$$

where $v_{kj}$ is the sample moment estimate of the variance, $h_{k}$ and $h_{h}$ are bandwidths for the variables and $\kappa_{2}$ is the variance of the kernel function for each variable. We used the rule of thumbs bandwidth, $h_{k}=1.06\sigma_{k}n^{-1/5},k=1,2$ and the product Gaussian kernel function where $\kappa_{2}=1$. The marginal and bivariate entropies of the ME model are computed using

(31) $$H\left(f^{*}\right)=1+\log\left(2\pi \right)+\frac{1}{2}\log\left|\Sigma \right|,$$

where |Σ| is the determinant of the covariance matrix of *f*. The entropies for the kernel PDF are obtained using the approximate formula (26) and the KL information divergence shown in the table is computed using (25). From these results we can see that the normal density is a reasonably good fit to the log transformed data.

#### 4.1.3. Disclosure Data and Inspections

We generate 400 pairs of data points from the ME model for disclosure and proceed with inspections to determine its quality as being a nontrivial replica of the actual data. Table 2 reports univariate and bivariate statistics for implementing the tasks 10–13 in Figure 1. The information moments of the two data sets are close to each other. Table 2 also gives the univariate and bivariate energy statistics and the fractions of univariate and bivariate Euclidean distances which are below 0.01. The energy statistics are at acceptable levels, according to 1000 simulations of the Cramér statistics [40]. Fractions of univariate and bivariate Euclidean distances which are below 0.01 are negligible.

**Figure 3:**
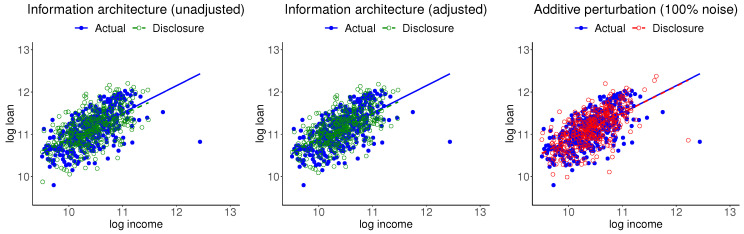
Scatter plots and regression lines of the actual and information architecture disclosure data with unadjusted and adjusted moments and disclosure data created by adding 100% noise and adjusted moments.

The left panel of Figure 3 shows the scatter plots of the actual and disclosure data, with the respective regression lines. It can be seen that the mass of actual data is close to the mass of the disclosure data, while there is not a one-to-one correspondence between the two sets. The actual data point with the highest income disappears in the disclosure data. The regression relationships, which are inclusive of all five information moments of the two data sets, are about the same.

Muralidhar et al. [13], in context of preserving confidentiality, considered including noise in the actual data and stipulated that marginal and joint summary measures of confidential attributes must be the same before and after perturbation. Moment adjustment is straightforward for this case. The middle panel of Figure 3 shows the corresponding plots for the moment-adjusted disclosure data. Features of this data are essentially the same as the unadjusted data. The right panel of Figure 3 shows the corresponding plots for the 100% noise disclosure data. While the mass of actual data is close to the mass of disclosure data, the noisy point corresponding to the actual data point with the highest income is clearly identifiable. (The noise added data shown here is chosen as a typical case from several replications of the process). One may include stronger noise, which raises the question of extensive noise domination. We should add that information moment-preserving transformation is straightforward when *T* consists of mean and covariances. This approach does not apply when *T* includes nonlinear functions or x is a nonlinear transformation of the original data y. Furthermore, Muralidhar et al. [13] also stipulated that confidential attributes must be the same before and after perturbation, but also noted that this feature does not hold beyond the normal distribution case. These issues limit applicability of this option to cases when the ME model is multivariate normal. We should also add that the first and second moments may not be defined for some important distributions like the Pareto distribution.

**Figure 4:**
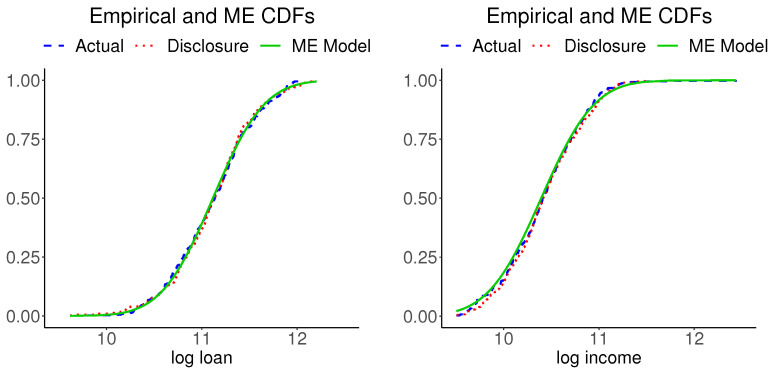
Empirical CDFs of the mortgage and disclosure data and the ME CDF of the actual data.

**Figure 5:**
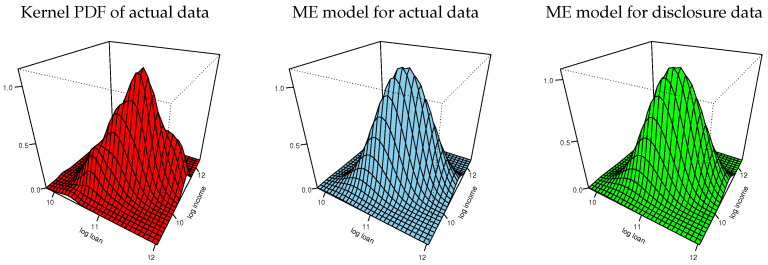
Bivariate kernel and ME densities of log-loan and log-income.

The information moments of disclosure data reported in Table 2 imply a bivariate normal distribution for implementing the tasks 14–16 in Figure 1. Figure 4 shows plots of the marginal CDFs of ME models for the actual data and the empirical CDFs of the actual data for each variable. The three CDFs of each variable are hardly distinguishable. (We also inspected the Kolmogorov–Smirnov distances between each pair of the respective variables in the actual and disclosure data and found them to be negligible). Figure 5 shows plots of the bivariate kernel PDF and bivariate PDFs for the two ME models. The kernel PDF looks like a rough version of the ME PDF plots and the ME PDFs are hardly distinguishable from each other.

Table 3 gives the results for the lower panel of Figure 1 which compare of the univariate and bivariate ME distributions for the actual and disclosure data. These results are obtained using known formulas for information measures of normal distribution. The mutual information is computed using the entropy Formula (17). This measure for the bivariate normal ME model can also be computed using the correlation coefficient

(32) $$M\left(X_{1},X_{2}\right)=-\frac{1}{2}\log\left(1-\rho^{2}\right),$$

which gives the normalized index $\delta^{2}\left(X_{1},X_{2}\right)=\rho^{2}$. The divergence between the two bivariate normal ME models is found by

(33) $$K\left(f^{**}:f^{*}\right)=\frac{1}{2}{\left(\mu_{1}-\mu_{2}\right)}^{\prime }\Sigma_{2}^{-1}\left(\mu_{1}-\mu_{2}\right)+\frac{1}{2}\left[\mathrm{Tr}\left(\Sigma_{1}\Sigma_{2}^{-1}\right)-\log|\Sigma_{1}\Sigma_{2}^{-1}|-2\right],$$

where the subscripts indicate the position of the PDFs in K(·,·) and Tr(·) denotes the trace. The results given in Table 3 suggest that the ME model for disclosure data preserves the statistical characteristics of the original data.

By the invariance of the KL divergence under one-to-one transformations, the information divergence and the mutual information measures reported in Table 3 apply to the data in the original dollar scale. However, the entropies shown in the table require adjustments according to entropy transformation formula. For $x_{k}=\log y_{k}$, the transformation formula gives

(34) $$\begin{array}{c} H\left(Y_{k}\right)=H\left(X_{k}\right)+\mu_{k},\;k=1,2, \end{array}$$

(35) $$\begin{array}{c} H\left(Y_{1},Y_{2}\right)=M\left(X_{1},X_{2}\right)+H\left(X_{1},X_{2}\right)+\mu_{1}+\mu_{2}. \end{array}$$

### 4.2. Bank Data

The data consists of sensitive variables such as the total amount of asset ($Y_{1}$) and the customer relationship score ($Y_{2}$), of the individuals which are considered here. These variables are important in financial modeling, but they also carry a risk of disclosure. Implementation of the information architecture is as follows.

#### 4.2.1. Exploratory Analysis

**Figure 6:**
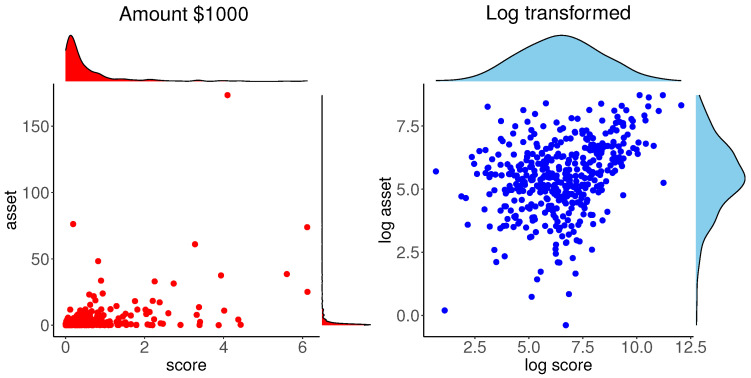
Plots of original and log transformed Bank data.

As in the previous example, our exploratory analysis of this data is depicted in Figure 6. The left panel shows the scatter plot of the data with the marginal PDFs obtained by the kernel density estimate. Mass of the data is concentrated near the origin and the marginal distributions are highly skewed. Like the mortgage data case, the marginal and bivariate plots of data suggest considering logarithm transformations of the variables. The scatter plot with the marginal PDFs for log transformed data are shown in the right panel of Figure 6. The PDFs of log transformed data are nearly symmetric, however, unlike the mortgage data case, this scatter plot is not elliptical. These plots suggest considering a non-elliptical bivariate distribution with symmetric marginals for the log transformed data $\left(x_{1},x_{2}\right)=\left(\log y_{1},\log y_{2}\right)$.

#### 4.2.2. Information Moments and ME Model

We consider a bivariate logistic model for the log transformed data which is the location-scale transformation of the standard bivariate logistic distribution given in Table A3; the location and scale parameters of the tabulated model are $\mu_{k}=0$ and $\lambda_{k}=1,k=1,2$, respectively. Table A3 gives the following information moments:

(36) $$\begin{array}{ccc} & & \int x_{k}f\left(x\right)dx=0,\;k=1,2, \end{array}$$

(37) $$\begin{array}{ccc} & & \int \int \log\left(1+e^{-x_{1}}+e^{-x_{2}}\right)f\left(x_{1},x_{2}\right)dx_{1}dx_{2}=\theta_{3}. \end{array}$$

We use the location-scale transformation of the standard logistic model and obtain its information moments by applying the location-scale transformation shown in Table A2.

The data information moments are computed using the means of log-transformed variables and the following relationship between the scale parameters and the standard deviations $\sigma_{k}$ of the logistic distribution:

(38) $$s_{k}=\frac{\sqrt{3}\sigma_{k}}{\pi },k=1,2.$$

Using this relationship gives $s_{1}=1.045$ and $s_{2}=0.798$ for the log-transformed Asset and Score, respectively. (The location and scale parameters can also be estimated by the maximum likelihood method, which gives similar values).

Table 4 reports univariate and bivariate statistics for implementing the tasks shown in the upper panel of Figure 1. The first column gives the information moments of the log-transformed data and the second column gives the corresponding parameters of the kernel estimate. The corresponding measures are close.

The marginal and bivariate entropies of the logistic ME model are obtained using

(39) $$H\left(X_{1},X_{2}\right)=4.5+\log\frac{s_{1}s_{2}}{2},\;H\left(X_{k}\right)=2+\log x_{k},\;k=1,2.$$

The entropies for the kernel PDF are computed using the approximate Formula (26) and the KL information divergence shown in the table is computed using (25). From these results we can see that the logistic density is a reasonably good fit to the log transformed data.

#### 4.2.3. Disclosure Data and Inspections

We generate 416 pairs of data points from the logistic ME model for disclosure and proceed with inspections to determine its quality as being a nontrivial replica of the actual data. Table 5 reports univariate and bivariate statistics for implementing the tasks 10–13 in Figure 1. The information moments of the two data sets are close to each other. In addition, the logistic scale parameters of the disclosure data for the Asset and Score are $s_{1}=1.126$ and $s_{2}=0.908$, respectively, which are close to their counterparts for the actual data. Table 5 also gives the univariate and bivariate energy statistics and the fractions of univariate and bivariate Euclidean distances which are all below 0.01. The energy statistics are at acceptable levels and fractions of univariate and bivariate Euclidean distances which are below 0.01 are all negligible.

**Figure 7:**
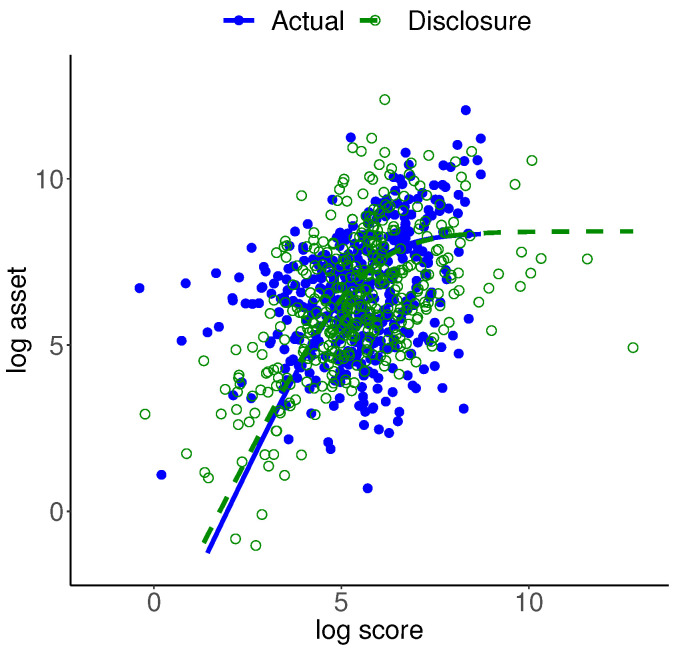
Scatter plots of the actual and disclosure data.

Figure 7 shows the scatter plots of the actual and disclosure data superimposed by the regression function of the bivariate logistic distribution for each data set. It can be seen that the mass of actual data is close to the mass of disclosure data, while there is not a one-to-one correspondence between the two sets. The expression for the regression is nonlinear, given as follows by Kotz et al. [46]:

(40) $$E\left(X_{k}|x_{h}\right)=\mu_{h}+s_{h}-s_{h}\log\left(1+\exp\left\{-\frac{x_{k}-\mu_{k}}{s_{k}}\right\}\right).$$

The plotted regression functions are inclusive of the three information moments and two scale parameters for each data set. In each case, we assessed the regression fit by its mean absolute error. This measure for the actual data is 1.817 and for the disclosure data is 1.486. In spite of the fact that the disclosure data is generated from the bivariate logistic distribution, we can conclude that the fit of the regression for the actual data is satisfactory. (We attempted implementing the nonlinear least squares function in R with the initial values of four regression parameters a+blog(1+exp{−(x−c)/d}) set as the actual data location and scale parameters. It failed due to the gradient singularity).

**Figure 8:**
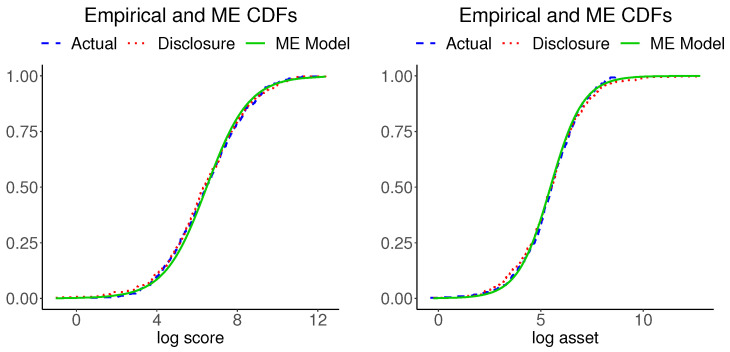
Empirical CDFs of the bank and disclosure data and the ME CDF of the actual data.

**Figure 9:**
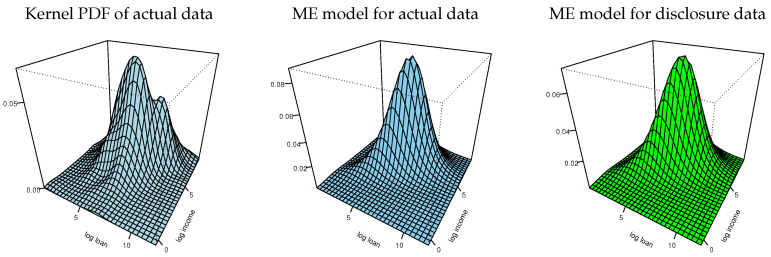
Bivariate kernel and ME densities of log-asset and log-score.

Figure 8 shows plots of the marginal CDFs of ME models for the actual data and the empirical CDFs of the actual data for each variable. The three CDFs of each variable are hardly distinguishable. (We also inspected the Kolmogorov–Smirnov distances between each pairs the respective variables in the actual and disclosure data and found them to be negligible). Figure 9 shows plots of the bivariate kernel PDF and bivariate PDFs for the two ME models. The kernel PDF seems a bumpy version of the ME PDF plots, as in our previous example, and the ME PDFs are hardly distinguishable from each other.

Table 6 gives the results for the lower panel of Figure 1 which compare of the univariate and bivariate ME distributions for the actual and disclosure data. These results are obtained using known formulas for information measures of logistic distribution. The mutual information for the bivariate logistic ME model is given by

(41) $$M(X_{1},X_{2})=\log2-0.5.$$

This constancy is due to the facts that this logistic distribution only includes the parameters of marginal distributions and the mutual information is invariant under one-to-one transformations of each variable, in particular, location-scale transformations, which makes the logistic distribution free from the location and scale parameters. Under the bivariate logistic distribution, dependence between $X_{1}$ and $X_{2}$ are imposed through the bivariate information moment (37). (A more general version of bivariate logistic includes an additional parameter. For the general logistic model the entropies and mutual information are functions of the additional parameter. The more common logistic model used in this paper is the special case of the general model with the additional parameter set to one).

Explicit expression for the KL divergence between two logistic distributions is not available. The divergence in Table 6 is found by the approximate Formula (25). The results in Table 6 suggest that the ME model for disclosure data preserves the statistical characteristics of the original data.

## 5. Concluding Remarks

The information architecture for the data disclosure problem proposed in this paper combines basic elements of statistics and information theory to produce statistical replicas of the actual data for disclosure. The architecture begins with an exploratory data analysis where statistical graphics and variable transformations summarize the statistical features of the data distribution. The numerical summaries are formulated in terms of information moments and a density plot is chosen as a graphical representation of the data. The information-theoretic approach uses the numerical summaries as partial information to provide the ME probability model for the unknown data-generating distribution. The data density plot is used as an input to the Kullback–Leibler information divergence for inspecting suitability of the information moments for representing the data-generating probability distribution. Upon approval in this first inspection, the ME model is used to generate statistical replicas of the actual data. Then the generated data is used to inspect the compatibility of its empirical CDF with the empirical CDF of the actual data. Further inspections include Euclidean distance measures between the disclosure and actual data points, Euclidean distance measures between the disclosure and actual data information moments and the Kullback–Leibler information divergence between the ME models for the disclosure and actual data. Disclosure data is ready for release upon approval of all inspections of the reliability of the disclosure data as a statistical replica of the actual data.

Implementation of the information architecture is illustrated using two financial data sets: Mortgage data and bank account data. Two variables from each data set are selected for illustrations. The distributions of variables in both data sets were highly skewed, which suggested subjecting data to logarithm transformations. The scatter plot of the log transformed mortgage variables was approximately elliptical. The KL information divergence confirmed suitability of the ME bivariate normal model for the log transformed data (lognormal model for the data), which was used to generate data for disclosure. Discrepancy between the underlying distributions of actual and disclosure data was inspected using energy statistic (a Euclidean distance-based measure) and information divergence. In addition, pairwise Euclidean distances between disclosure data points and actual data points were inspected for the disclosure risk.

Like the mortgage variables, log transformations of the variables in bank data induced near symmetrical distributions for the variables. However, unlike the case of the mortgage data, the scatter plot of the variables in bank data was not elliptical. These conditions suggested a non-elliptical symmetric bivariate distribution. We considered the ME bivariate logistic model for this data. The model and disclosure data were inspected according to the proposed information architecture. This example illustrated that, like producing a portrait, several attempts may be needed for generating a set of disclosure data for an acceptable replica of the actual data.

Our illustrations were limited to two variables. Implementation of the information architecture for cases where the data features suggest information moments for multivariate ME models with all marginal distributions in the same family are rather straightforward. Examples include the normal, logistic, Pareto and Dirichlet. In our bivariate examples we started with the univariate cases and built up to the bivariate model and reported inspection results for the univariate and bivariate models. This approach can be used in higher dimensions. In Section 3, we noted that link functions such as copula can be used for cases where the univariate marginal distributions are in different families.

The purpose of information moments and the implied ME model is to produce a statistical copy of the actual data such that both data sets possess approximately the same statistical information. A set of information moments can fail to accomplish the purpose. Jaynes [47] recalls a historical fact that the “seemingly ‘unsuccessful’ application of the principle of maximum entropy” by Gibbs provided clues about development of new theories in statistical mechanics and quantum physics. Using a combinatoric argument and an asymptotic he shows that “the distribution predicted by maximum entropy can be realized experimentally in overwhelmingly more ways than can any other” that satisfies the information moment condition. He points out the following weaker implication:

“If the information incorporated into the maximum-entropy analysis includes all the constraints actually operative in the random experiment, then the distribution predicted by maximum entropy is overwhelmingly the most likely to be observed experimentally, because it can be realized in overwhelmingly the greatest number of ways.

Conversely, if the experiment fails to confirm the maximum-entropy prediction, and this disagreement persists on indefinite repetition of the experiment, then we will conclude that the physical mechanism of the experiment must contain additional constraints which were not taken into account in the maximum-entropy calculations. The observed deviations then provide a clue as to the nature of these new constraints.”

(Jaynes [47])

In the context of information architecture, disagreement between the actual and disclosure data sets is inspected multiple times. If disagreement between the two data sets persists on repetitions of generating the statistical copy, then we conclude that the distribution that generated the actual data must contain additional constraints which were not taken into account in the calculation of the ME model.

In the proposed information architecture, the information is drawn through exploring the distributional features of the data for application of the ME principle. Statistical modeling of data is said to involve a combination of art (visualizing information from the data) and science (knowledge of data generating process, mathematical representation of data). Selecting a set of information moments for consideration requires familiarity with properties of varieties of probability distributions. The data structures of our illustrative examples enabled us to identify ME models rather easily. Identifying a known ME distribution for data with more complex structures can be more difficult or impossible. For such cases, new ME models that draw numerous information moments should be developed. For example, Bajgiran et al. [31] showed that the ME model with a set of quantile information in finite range is the piecewise uniform distribution (a histogram). To implement such a basic model in the information architecture, quantile information for each variable can be drawn from the data and combined by a link function, such as a copula, to obtain a model for the entire data.

The information theoretic framework proposed here for the data disclosure problem provides unique research opportunities to develop multivariate generalizations and statistical inference procedures including Bayesian methods. Both entropy and Kullback–Leibler information are instrumental in the proposed architecture and in our set-up we have considered the use of classical methods for estimation of these quantities as well as in the implementation of the ME framework. The ME framework requires nonparametric estimation of the data distribution and nonparametric Bayesian methods can be used for this purpose. Earlier Bayesian works in this area include [48], who proposed the use of Dirichlet process priors in estimation of nonparametric entropy and Kullback–Leibler information in the ME framework. The development presented by these authors relies on a quantized approximation of entropy for the univariate case. Extension of their methodology to the multivariate case poses challenging methodological and computational issues.

## Figures and Tables

**Table 1:** Information moments of log-transformed mortgage data and kernel PDF and information divergence between the kernel and ME PDFs.

	Information Moment		Entropy		KL Divergence	K Index	Coin
	Actual	Kernel	$H(f^{*})$	$H(\tilde{f})$	$K(\tilde{f}:f^{*})$	$\delta^{2}\left(K\right)$	q(K)
Loan			0.563	0.564	0.009	0.017	0.565
Mean	11.117	11.111					
Variance	0.180	0.189					
Income			0.594	0.609	0.016	0.031	0.588
Mean	10.394	10.389					
Variance	0.192	0.203					
Bivariate			0.925	0.866	0.072	0.134	0.683
Covariance	0.123	0.118					

**Table 2:** Information moments and Euclidean measures for log-transformed mortgage data and disclosure data.

	Information Moment		Energy Stat	Euclidean Dist
	Actual	Disclosure	$E(x,x^{*})$	$\pi_{d}\left(x_{i},x_{h}^{*}\right)<0.01$
Loan			0.134	0.027
Mean	11.117	11.115		
Variance	0.180	0.188		
Income			0.065	0.026
Mean	10.394	10.397		
Variance	0.192	0.191		
Bivariate			0.201	<0.001
Covariance	0.123	0.119		

**Table 3:** Information measures of the ME models for the mortgage data and disclosure data.

	Entropy		KL Divergence	K Index	Coin
	$H(f^{*})$	$H(f^{**})$	$K(f^{**}\;:f^{*})$	$\delta^{2}\left(K\right)$	q(K)
Loan	0.563	0.583	<0.001	0.001	0.514
Income	0.595	0.593	<0.001	<0.001	0.504
Bivariate	868	0.923	0.004	0.007	0.542
Mutual info	0.290	0.253			
M index	0.440	0.397			
Coin index	0.832	0.815			

**Table 4:** Information moments of log-transformed bank data and kernel PDF and information divergence between the kernel and ME PDFs.

	Information Moment		Entropy		KL Divergence	K Index	Coin
	Actual	Kernel	$H(f^{*})$	$H(\tilde{f})$	$K(\tilde{f}:f^{*})$	$\delta^{2}\left(K\right)$	q(K)
Asset			2.044	2.085	0.016	0.031	0.589
Mean	6.473	6.461					
Score			1.774	1.787	0.014	0.027	0.582
Mean	5.470	5.457					
Bivariate			3.625	3.766	0.283	0.432	0.828
Log-sum-expo	1.161	1.518					

**Table 5:** Information moments and Euclidean measures for log-transformed bank data and disclosure data.

	Information Moment		Energy Stat	Euclidean Dist
	Actual	Disclosure	$E(x,x^{*})$	$\pi_{d}\left(x_{i},x_{h}^{*}\right)<0.01$
Asset			0.460	0.006
Mean	6.473	6.376		
Score			0.655	0.008
Mean	5.470	5.481		
Bivariate			2.529	<0.001
Log-sum-expo	1.161	1.495		

**Table 6:** Information measures of the ME models for the bank data and disclosure data.

	Entropy		KL Divergence	K Index	Coin
	$H(f^{*})$	$H(f^{**})$	$K(f^{**}\;:f^{*})$	$\delta^{2}\left(K\right)$	q(K)
Asset	2.044	2.119	0.005	0.010	0.550
Score	1.774	1.903	0.009	0.018	0.568
Bivariate	3.625	3.829	0.002	0.005	0.535
Mutual info	0.193	0.193			
M index	0.320	0.320			
Coin index	0.783	0.783			

## Data Availability

The disclosure data used in this study is available from the authors.

## Appendix A

The following three tables list examples of univariate and bivariate ME entropy distributions and their information moments.

**Table A1:** Examples of univariate maximum entropy models and information moments.

ME Model	Density	Information Moments
Generalized error, x∈ℜ (Laplace β=1, Normal β=2)		
	$f\left(x\right)=\frac{\beta }{2\sigma \Gamma (1/\beta )}e^{-{\left|\frac{x-\mu }{\sigma }\right|}^{\beta }}$	$\left\{\begin{array}{c} T_{1}\left(x\right)=x,\\ T_{2}\left(x\right)={\left|x-\mu \right|}^{\beta } \end{array}\right.$
Student-*t*, (Cauchy ν=1), x∈ℜ		
	$f\left(x\right)=\frac{\Gamma (\nu /2+1/2)}{\sqrt{\nu \pi }\Gamma \left(\nu /2\right)}{\left(1+\frac{x^{2}}{\nu }\right)}^{-\frac{\nu +1}{2}}$	$T\left(x\right)=\log\left(\nu +x^{2}\right)$
Logistic, x∈ℜ		
	$f\left(x\right)=\frac{e^{-x}}{{\left(1+e^{-x}\right)}^{2}}$	$\left\{\begin{array}{c} T_{1}\left(x\right)=x,\\ T_{2}\left(x\right)=\log\left(1+e^{-x}\right) \end{array}\right.$
Asymmetric Laplace, x∈ℜ		
	$f\left(x\right)=\left\{\begin{array}{cc} \frac{\lambda }{1/a+1/b}e^{-\lambda c_{o}\left(q-x\right)} & x\leq q\\ \frac{\lambda }{1/a+1/b}e^{-\lambda c_{u}\left(x-q\right)} & x>q \end{array}\right.$	$\left\{\begin{array}{cc} a(q-x), & x\leq q\\ b(x-q), & x>q, \end{array}\right.$
Exponential [Exp(β)],x≥0		
	$f\left(x\right)=\lambda e^{-\lambda x}$	T(x)=x
Pareto Type II [ParII(α)], x≥0		
	$f\left(x\right)=\frac{\alpha }{{\left(1+x\right)}^{-\alpha -1}}$	T(x)=log(1+x)
Gamma [G(α,β)], x≥0		
	$f\left(x\right)=\frac{\beta^{\alpha }}{\Gamma (\alpha )}x^{\alpha -1}e^{-\beta x}$	$\left\{\begin{array}{c} T_{1}\left(x\right)=x,\\ T_{2}\left(x\right)=\log x \end{array}\right.$
Beta [Beta(α,β)], x∈[0,1]		
	$f\left(x\right)=\frac{1}{B(\alpha ,\beta )}x^{\alpha -1}{\left(1-x\right)}^{\beta -1}$	$\left\{\begin{array}{c} T_{1}\left(x\right)=\log x,\\ T_{2}\left(x\right)=\log\left(1-x\right) \end{array}\right.$

**Table A2:** Examples of univariate maximum entropy models obtained by transformation and information moments.

Family and Transformation	Density	Information Moments
Location-scale transformation		
Y=σX+μ	$f_{y}\left(y\right)=\frac{1}{\sigma }f_{x}\left(\frac{y-\mu }{\sigma }\right)$	$T_{j}\left(y\right)=T_{jx}\left(\frac{y-\mu }{\sigma }\right)$
Log and exponential transformations		
Logistic, y∈ℜ Y=−logX, X∼Par II(1)		
	$f\left(y\right)=\frac{e^{-y}}{{\left(1+e^{-y}\right)}^{2}}$	$\left\{\begin{array}{c} T_{1}\left(y\right)=y,\\ T_{2}\left(y\right)=\log\left(1+e^{-y}\right) \end{array}\right.$
Log-Gamma, y∈ℜ,α,β>0 Y=logX,X∼G(α,β)		
	$f\left(y\right)=\frac{\beta^{\alpha }}{\Gamma (\alpha )}e^{\alpha y}e^{-\beta e^{y}}$	$\left\{\begin{array}{c} T_{1}\left(y\right)=y,\\ T_{2}\left(y\right)=e^{y} \end{array}\right.$
Lognormal, y>0,,μ∈ℜ,σ>0 $Y=e^{X},X∼N\left(\mu ,\sigma^{2}\right)$		
	$f\left(y\right)=\frac{1}{\sqrt{2\pi }\sigma y}e^{-\frac{{\left(\log y-\mu \right)}^{2}}{2\sigma^{2}}}$	$\left\{\begin{array}{c} T_{1}\left(y\right)=\log y,\\ T_{2}\left(y\right)={\left(\log y-\mu \right)}^{2} \end{array}\right.$
Power transformations		
Generalized Gamma, y>0,α,τ,β>0 $Y=X^{1/\tau },X∼G\left(\alpha ,\beta \right)$ (Weibull α=1, Half-normal α=1/2,τ=2), Generalized normal τ=2		
	$f\left(y\right)=\frac{\beta^{\alpha \tau }}{\Gamma (\alpha )}y^{\alpha \tau -1}e^{-{\left(\beta y\right)}^{\tau }}$	$\left\{\begin{array}{c} T_{1}\left(y\right)=y^{\tau },\\ T_{2}\left(y\right)=\log y \end{array}\right.$
Pareto Type IV, y≥0,α,τ>0 $Y=X^{1/\tau },X∼ParII\left(\alpha \right)$ (α=1 Pareto Type III)		
	$f\left(y\right)=\frac{\alpha \tau y^{\tau -1}}{{\left(1+y^{\tau }\right)}^{\alpha +1}}$	$\left\{\begin{array}{c} T_{1}\left(y\right)=\log y,\\ T_{2}\left(y\right)=\log\left(1+y^{\tau }\right) \end{array}\right.$
Inverted beta, y≥0,α,β>0 $Y=X^{-1},X∼Beta\left(\alpha ,\beta \right)$		
	$f\left(y\right)=\frac{1}{B(\alpha ,\beta )}\frac{y^{\beta -1}}{{\left(1+y\right)}^{\alpha +\beta }}$	$\left\{\begin{array}{c} T_{1}\left(y\right)=\log y,\\ T_{2}\left(y\right)=\log\left(1+y\right) \end{array}\right.$

**Table A3:** Examples of bivariate maximum entropy models and information moments.

ME Model	Density	Information Moments
$\begin{array}{c} \;\mathrm{Normal}\;x\in {ℜ}^{2}\\ \;X_{i}∼N\left(\mu_{i},\sigma_{i}^{2}\right),\;\left(\mathrm{Similar}\;\mathrm{multivariate}\;\mathrm{case}\right) \end{array}$		
$\;\;\;\;f\left(x\right)=\frac{1}{{2\pi |\Sigma |}^{1/2}}\exp\left\{-\frac{1}{2}{\left(x-\mu \right)}^{\prime }\Sigma^{-1}\left(x-\mu \right)\right\}$		$\left\{\begin{array}{c} T_{1}\left(x\right)=x_{1},T_{2}\left(x\right)=x_{2}\\ T_{3}\left(x\right)=x_{1}^{2},T_{4}\left(x\right)=x_{2}^{2}\\ T_{5}\left(x\right)=\left(x_{1}-\mu_{1}\right)\left(x_{2}-\mu_{2}\right) \end{array}\right.$
$\begin{array}{c} \;\mathrm{Logistic}x_{i}\in {ℜ}^{2}\\ \;X_{i}∼Logist\left(0,1\right),\left(\mathrm{Similar}\;\mathrm{multivariate}\;\mathrm{case}\right) \end{array}$		
$\;\;\;\;f\left(x\right)=\frac{2e^{-x_{1}-x_{2}}}{{\left(1+e^{-x_{1}}+e^{-x_{2}}\right)}^{3}}$		$\left\{\begin{array}{c} T_{1}\left(x\right)=x_{1},T_{2}\left(x\right)=x_{2}\\ T_{3}\left(x\right)=\log\left(1+x_{1}+x_{2}\right) \end{array}\right.$
$\begin{array}{c} \;\mathrm{Farlie-Gumbel-Morgenstern}\;(\mathrm{F-G-M}),x_{i}\in \left[0,1\right]\\ \;X_{i}∼Uniform,\left(\mathrm{Similar}\;\mathrm{multivariate}\;\mathrm{case}\right) \end{array}$		
$\;\;\;\;f\left(x\right)=1+\alpha \left(1-2x_{1}\right)\left(1-2x_{2}\right)$		$T\left(x\right)=\log[1+\alpha \left(1-2x_{1}\right)\left(1-2x_{2}\right)]$
$\begin{array}{c} \;\mathrm{Dirichlet}x\in {\left[0,1\right]}^{2},\alpha_{1},\alpha_{2},\alpha_{3}>0\\ \;X_{i}∼Beta\left(\alpha_{i},\alpha_{j}+\alpha_{3}\right),\left(\mathrm{Similar}\;\mathrm{multivariate}\;\mathrm{case}\right) \end{array}$		
$\;\;\;\;f\left(x\right)=\frac{\Gamma (\alpha_{1}+\alpha_{2}+\alpha_{3})}{\Gamma \left(\alpha_{1}\right)\Gamma \left(\alpha_{2}\right)\Gamma \left(\alpha_{3}\right)}x_{1}^{\alpha_{1}-1}x_{2}^{\alpha_{2}-1}{\left(1-x_{1}-x_{2}\right)}^{\alpha_{3}-1}$		$\left\{\begin{array}{c} T_{1}\left(x\right)=\log y_{x},T_{2}\left(x\right)=\log x_{2}\\ T_{3}\left(x\right)=\log\left(1-x_{1}-x_{2}\right) \end{array}\right.$
$\begin{array}{c} \;\mathrm{McKay’s}\;\mathrm{bivariate}\;\mathrm{gamma},\;0<x_{2}<x_{1},\alpha ,\;\beta ,\;\lambda >0\\ \;X_{1}∼G\left(\alpha +\beta ,\lambda \right),X_{2}∼G\left(\alpha ,\lambda \right) \end{array}$		
$\;\;\;\;f\left(x_{1},x_{2}\right)=\frac{\lambda^{\alpha +\beta }}{\Gamma (\alpha )\Gamma (\beta )}x_{2}^{\alpha -1}{\left(x_{1}-x_{2}\right)}^{\beta -1}e^{-\lambda x_{1}}$		$\left\{\begin{array}{c} T_{1}\left(x\right)=x_{1},\;T_{2}\left(x\right)=\log x_{2}\\ T_{3}\left(x\right)=\log\left(x_{1}-x_{2}\right) \end{array}\right.$
$\begin{array}{c} \;\mathrm{Gamma–gamma}\;\mathrm{mixture},x_{1},x_{2}\geq 0,\alpha ,\beta ,\lambda_{1},\lambda_{2}>0\\ \;X_{1}∼G\left(\alpha ,\lambda_{1}\right),X_{1}X_{2}∼G\left(\beta ,\lambda_{2}\right),X_{2}∼IB\left(\alpha ,\beta ,\lambda_{2}/\lambda_{1}\right)\\ \;(\mathrm{Gamma-exponential}\;\mathrm{mixture},\alpha =1) \end{array}$		
$\;\;\;\;f\left(x_{1},x_{2}\right)=\frac{\lambda_{1}^{\alpha }\lambda_{2}^{\beta }}{\Gamma (\alpha )\Gamma (\beta )},x_{1}^{\alpha +\beta -1}\;x_{2}^{\beta -1}e^{-\lambda_{1}x_{1}-\lambda_{2}x_{1}x_{2}}$		$\left\{\begin{array}{c} T_{1}\left(x\right)=x_{1},\\ T_{2}\left(x\right)=\log x_{1},\;T_{3}\left(x\right)=\log x_{2},\\ T_{4}\left(x\right)=x_{1}x_{2} \end{array}\right.$
